# Spatiotemporal Gait Asymmetries Remain Unaffected by Increased Load Carriage in Professional Intervention Police Officers

**DOI:** 10.3390/bioengineering11111140

**Published:** 2024-11-13

**Authors:** Davor Rožac, Mario Kasović, Damir Knjaz

**Affiliations:** 1 Department of Kinesiology of Sport, Faculty of Kinesiology, University of Zagreb, 10 000 Zagreb, Croatia; davor.rozac@kif.unizg.hr; 2 Department of General and Applied Kinesiology, Faculty of Kinesiology, University of Zagreb, 10 000 Zagreb, Croatia; 3 Department of Physical Activities and Health Sciences, Faculty of Sport Studies, Masaryk University, 625 00 Brno, Czech Republic; 4 Department of Sports Kinesiology, Faculty of Kinesiology, University of Zagreb, 10 000 Zagreb, Croatia; damir.knjaz@kif.hr

**Keywords:** specialized populations, standardized load carriage, asymmetry index, differences

## Abstract

Background: Although evidence indicates that load carriage may have an influence on walking patterns, the specific impacts of progressively increased loads on spatial and temporal gait asymmetries remain underexplored. Therefore, the primary aim of this study was to examine whether an increased load carriage had an effect on spatiotemporal gait asymmetries among intervention police officers. Methods: For the purpose of this study, 96 male intervention police officers were recruited and assessed under four load conditions: (i) “No load”, (ii) “a 5 kg load”, (iii) “a 25 kg load”, and (iv) “a 45 kg load”. Spatial and temporal gait parameters were measured using a pedobarographic platform (Zebris FDM). The spatial and temporal gait parameters, along with the ground reaction forces beneath different foot regions, were examined. The gait asymmetry for each parameter was calculated using the formula (x_right −_ x_left_)/0.5 × (x_right_ + x_left_)*100%, where “x” represents the numerical value of each parameter for the left and right sides of the body. Results: The findings indicated no statistically significant differences in the spatiotemporal parameters, nor ground reaction force gait asymmetries between the left and right foot, during walking under a progressively increased load carriage. Additionally, the parameter values for both the left and right sides of the body remained consistent, with a high intercorrelation observed across all of the loading conditions. The gait speed and ground reaction forces, which served as covariates, did not significantly change the spatiotemporal gait asymmetries. Conclusions: In summary, this study demonstrates that an increased load carriage did not lead to a progressive rise in spatiotemporal gait asymmetries in professional intervention police officers. However, further examination using an advanced 3-D gait analysis and an assessment of physiological patterns and adaptations is recommended to identify and confirm the key factors influencing gait asymmetry.

## 1. Introduction

Load carriage in specialized populations, such as police officers and their branches, has become a significant factor in ensuring protection and survival in high-risk situations [1,2]. Indeed, carrying an external load can provide the necessary resources for daily combat tasks and missions. Although the load has its benefits, the interaction among the individual, the load, and everyday duties and responsibilities is often associated with overall health and the quality of life [3,4]. Numerous studies have investigated the effects of carrying an external load on physiological and biomechanical changes [5,6,7]. From a physiological perspective, it is expected that heavier loads increase total and active energy expenditure, heart rate, and breathing characteristics [5] due to the greater forces applied on the musculoskeletal system. On the other hand, when it comes to the biomechanical approach, the literature has consistently reported increased flexion in the trunk, hip, and knee, as well as greater extension moments in the knee and ankle regions of the body [7]. However, there are limited data on spatial and temporal gait characteristics, as well as the differences between the sides of the body, in response to progressively heavier external loads; these have yet to be determined.

During bipedal movements, it is normal to observe a certain level of asymmetry, i.e., the inequality between the left and right sides of the body regarding a given parameter. The term “asymmetry” is one of the key factors and a starting point in defining optimal ergonomic efficiency and load positioning on the body. Thus, it is not surprising that it has gained significant attention over the last two decades [8,9,10]. Indeed, efforts have been made to determine an optimal level of asymmetry for certain physical performance parameters, indicating that a 15% difference between the sides of the body represents an upper threshold [9]. However, these patterns have rarely been observed in the lower extremities, where the literature indicates an increase of up to 50% when carrying heavier loads [8]. The majority of studies have attempted to examine the effects of uneven load carriage on gait biomechanics [10,11,12,13,14,15]. When carrying an asymmetrical load, previous kinematic analyses have shown that the body naturally increases extensor moments in the hip and knee of the unloaded leg [12]. While examining joint movement during walking, little is known about the relationship between load carriage and spatiotemporal gait asymmetries [11]. The available studies on this topic have shown an increase in gait asymmetry in the ground reaction forces in the medio-lateral direction when heavier loads (up to roughly 20% of the body’s weight) are applied [11]. Most recently, a study by Štefan et al. [16] indicated that a 3.5 kg load significantly increased asymmetries in the gait cycle, particularly during the stance, load response, single-limb support, pre-swing, and swing phases and in the step time, compared to the no-load condition in a large sample of police recruits. However, the limitation of the aforementioned study is its exclusive focus on a 3.5 kg load, while the effects of heavier loads on spatiotemporal gait asymmetries remain unexamined.

As the authors of this study are aware, only a handful of studies have attempted to examine the effects of a gradually increasing load carriage on spatiotemporal gait asymmetries. From a practical standpoint, establishing the potential increases in gait asymmetry may result in the even greater eversion and external rotation of the foot, decreases in the step and stride length, and increases in the step and stride time [17], which could lead to injuries and stress fractures [18,19,20].

Given the critical role of symmetrical gait in performing daily tasks and assignments, it is reasonable to propose that the addition of weight may alter the gait parameters—both temporally and spatially—and have an immediate impact on gait asymmetries. Such disparities in the values between the left and right sides of the body may lead to an altered posture, a decline in function, and an increased risk of injury. However, analyzing how body asymmetry varies under specific loading conditions may provide valuable insights for rearranging or restructuring the current loads to reduce the risk of harmful biomechanical impacts on the body during walking. Lastly, public health policymakers should gain a better understanding of load safety concerns and the potential applications of the findings.

Therefore, the primary aim of this study was to examine whether an increased load carriage had effects on spatiotemporal gait asymmetries among intervention police officers. We hypothesized that gait asymmetries would gradually increase with the greater load carriage.

## 2. Materials and Methods

### 2.1. Study Participants

This observational study was conducted among male officers of the Zagreb Police Department Police Intervention Unit, who were anonymously tested. The sample size was calculated using the G*Power (version 3.1.9.7 GmbH) statistical calculator, with a statistical power of 0.80, *p* < 0.05, and a large effect size (0.40), which resulted in a sufficient sample size of N = 80 participants. Considering the dispersal of the sample during the study’s implementation, an additional 20% increase was added to the sample size, resulting in N = 96. All of the subjects recruited for the study had been employees of the Police Intervention Unit for at least three years. All of the participants before and during testing were healthy and had no acute chronic illnesses or disabilities that could prevent their participation in the research or lead to the termination of their participation. Before conducting the study, written informed consent was obtained from all of the participants. The study procedure and the testing protocol were approved by the Ethical Committee of the Faculty of Kinesiology and the Police Intervention Department under the Ministry of Internal Affairs of the Republic of Croatia (Ethical code: 511-01-128-23-1).

### 2.2. Loading Conditions

Each test subject crossed a platform while bearing one of four standard loads recommended by the Ministry of Internal Affairs for intervention police officers. The first load was body-weight-only (referred to as “No load”); the second was a 5 kg load (referred to as “Load 1”), which consisted of a belt with a pistol that was loaded with a full handgun magazine, an additional full handgun magazine, and handcuffs; the mean weight of all of the participants was ±SD = 4.97 ± 0.25 kg. The third load was a 25 kg load (referred to as “Load 2”), which was upgraded with a helmet, a ballistic vest, and a multipurpose baton. The fourth load was a 45 kg load (referred to as “Load 3”), which was upgraded with additional lower extremity protection and a protective gas mask (or ±SD = 45.10 ± 4.33 kg). The load position in the second loading condition was around the participant’s hip; for the third load, the helmet was placed on the head, while a ballistic vest was positioned on the chest region, with a multipurpose baton in front of the trunk; for the final, fourth condition, the protection was placed on the knees and arms and a protective gas mask was put behind the head. The sequence of each load was randomized to reduce the impact of a learning effect [21].

### 2.3. Spatiotemporal Gait Parameters

In order to determine the spatial and temporal parameters, we used the ZEBRIS FDM 1.12. software, which produced data following each attempt. The laptop and software were connected to the platform and set up on the computer, providing immediate data on the gait biomechanics. Spatial and temporal gait parameters were created and pre-programmed within the software. For example, the software recorded spatial measurements such as foot rotation (°), step length (cm), step width (cm), the length of the gait line from first to final foot contact with the ground (mm), and a single limb support line (mm). The degree of foot rotation was determined by measuring the angle between the foot’s position and the line connecting both feet. Step length refers to the distance between the heel of one foot and the heel of the other foot, while stride length combines the distances of both steps. The temporal parameters consisted of step durations (s). Step time was defined as the duration between the heel strikes of both feet upon contact with the ground. Gait speed was presented in km/h. Additionally, other temporal parameters were expressed as a percentage of the gait cycle for each foot: the stance phase, which included the load response; mid-stance; pre-swing; and the swing phase. It should be noted that foot rotation, step length, the length of the gait line from initial to final foot contact, the single support line, step time, and the percentage of the gait cycle were measured for both the left and right feet. The ground reaction forces for both feet beneath the forefoot, midfoot, and hindfoot regions were calculated and are presented in N.

### 2.4. Testing Procedure

In order to measure the spatiotemporal gait parameters, we utilized a pedobarographic platform from the ZEBRIS company, FDM, GmbH, Munich, Germany, which was equipped with 11,264 sensors that were operating at a sampling rate of 100 Hz and had a sensor area of 149 cm × 54.2 cm. This tool is user-friendly for studying gait characteristics, and we followed a testing procedure similar to that used in previous studies [21]. During one day of measuring all four of the levels of equipment for the members of the intervention police, we demonstrated the operation of the system and data collection to the respondents. The method of walking across the platform was explained to everyone, and it was noted that the selection of standard police equipment in different variants was chosen by random selection. Also, none of the respondents had participated in similar research, and, therefore, the effect of learning or adapting to the measurement process was avoided. Each participant carried a randomly selected load across the platform, after which, the procedure of randomly selecting equipment and walking across the platform was repeated. Two custom-built wooden platforms were positioned before and after the testing area to facilitate normal walking. Upon receiving a signal from the researcher, the participants began to move across the platform. Upon reaching the end of the walkway, the participants stopped, turned around, and headed back to their starting position. After measuring each load by randomly selecting each subject, the raw data were automatically entered into the data matrix. An analysis of cross-correlation across all eight trials demonstrated outstanding reliability (r > 0.90).

### 2.5. Statistical Analysis

To assess data normality, we employed the Kolmogorov–Smirnov test. The variables that followed normal distribution were presented as the mean and the standard deviation (SD), and the variables that were not normally distributed were presented as the median and the interquartile range (25th–75th). Asymmetries between the different loading conditions were tested using a one-way repeated measures ANOVA or the Friedman test. If a significant *p*-value was generated, a Bonferroni *post-hoc* test between the loading conditions was applied. The effect size was presented as the partial eta squared, with the following values: “small” (0.01), “medium” (0.06), and “large” (0.14). The gait asymmetries were calculated using the formula proposed by Robinson et al. [22]: (x_right_ − x_left_)/0.5*(x_right_ + x_left_)*100%, where “x” represents a given parameter being calculated. A score of 0 denotes a perfectly symmetrical gait, while an increasing value in either the positive or negative direction indicates a greater asymmetry. Of note, the right side of the body was chosen habitually, and therefore, the values for the left and right sides of the body were entered into the equation. The statistical significance was set at a priori *p* ≤ 0.05. All of the analyses were performed using the Statistical Package for Social Sciences (SPSS Inc., Chicago, IL, USA).

## 3. Results

Basic descriptive statistics and the changes between the left and right sides of the body under different loading conditions in the spatial gait parameters are presented in Table 1. Carrying heavier loads did not result in significant changes to the spatial gait parameters, indicating that the participants maintained similar gait patterns while carrying these loads. Notably, the intercorrelation between the loading conditions for foot rotation, step length, the length of the gait line, and the single limb support line was r > 0.90, with the coefficient of variation (CV) being <1.5% within every loading condition, indicating that the participants exhibited similar spatial values and gait biomechanics. When comparing the sides of the body, the mean difference between the left and right foot was not statistically significant, which was also observed for the symmetry index. Heavier loads did not produce a greater asymmetry in the spatial gait parameters, although more substantial changes in asymmetry were observed for the single limb support line, which approached statistical significance. Foot rotation remained the most stable spatial parameter of gait across the different loading conditions.

Changes in the temporal gait parameters are shown in Table 2. Similarly to the spatial gait parameters, no significant changes in any of the measured parameters were observed, irrespective of the side of the body. The intercorrelation coefficient was also extremely high (r > 0.90, CV < 2.0%) within each side of the body. When comparing the sides of the body, the mean difference between the left and the right foot was not statistically significant, which was also observed for the symmetry index. Additionally, heavier loads did not produce a greater asymmetry in the temporal gait parameters, although more substantial changes in asymmetry were observed for the stance, load response, and swing phases of the gait.

Table 3 shows changes in the ground reaction force asymmetries following the different loading conditions. The findings indicated no significant changes in the force asymmetries beneath the different foot regions when a gradually heavier load was added. Of note, when each model was adjusted for gait speed and ground reaction force, similar patterns and effect sizes remained.

## 4. Discussion

The main purpose of this study was to examine the effects of an increased load carriage on spatiotemporal gait asymmetries in intervention police officers. The findings of the study indicate no significant differences between the left and right sides of the body, nor any expected increases in the spatial and temporal gait asymmetries following the addition of a gradually heavier load.

To the authors’ knowledge, thus far, no studies have explored changes in spatial and temporal gait asymmetries under different loading conditions in intervention police officers. One common approach in detecting gait imbalances typically involves measuring the ground reaction forces between the feet and the ground during a stance position [11,23]. It has been shown that approximately two-thirds of the participants exhibited greater foot asymmetry in the transversal and frontal planes, compared to when carrying no load. However, limited data exist on examining the same patterns during walking. When comparing the sides of the body regarding the ground reaction forces during walking, a study by Zhang et al. [11] found that heavier loads led to a greater asymmetry index, but the same load did not affect both feet equally.

Although we hypothesized that heavier external loads would gradually produce greater spatial and temporal gait asymmetries, we did not observe such findings. One potential mechanism could be attributed to a learning effect and the participants’ experience in carrying such loads on a daily basis. For example, of the three loads studied in this research, the first is often carried throughout the day, typically for 8 to 10 h. The second level of equipment is commonly used in urban situations (such as maintaining order at soccer matches, etc.), which require a higher level of risk and an additional level of protection. On average, such equipment is worn two to three times a week for approximately 12 h. The third level of equipment is intended for tasks involving the control of immigrants, terrorist attacks, etc. Intervention police officers wear this equipment for durations of 10 days to 2 weeks, for approximately 10 to 12 h per day. Given the regular use of all of the levels of equipment and the extensive service experience of the intervention police officers, it is reasonable to assume that they have adapted to wearing heavier official gear, which does not significantly alter their walking patterns, particularly in the area of spatial and temporal parameters. Although we did not perform a 3-D analysis of the upper extremities, it is speculated that certain adjustments were made in the inertial patterns of the musculoskeletal system due to the load placement on the body, which may have limited the natural arm swing during walking.

Despite non-significant changes in asymmetry with heavier loads, the evidence implies that the trunk tends to lean away from the side carrying the load, suggesting that motor control responses to external loads may be related to load-carrying strategies and characteristics. Differences in posture between the left and right sides are influenced by the dominant side of the body, which directly affects gait asymmetry throughout the kinetic chain. Although asymmetry often occurs because of variations in stride length or cadence/walking speed [5], the findings of this study did not reveal significant asymmetry changes in these parameters. To overcome this problem, we adjusted for gait speed and ground reaction forces, which may interact with spatial and temporal gait parameters following different loading conditions. However, we found no significant interaction effect of the aforementioned covariates in any of the models, indicating that neither gait speed nor ground reaction forces significantly affected the gait with the heavier loads. The reason for this result may be the relatively homogenous sample of intervention police officers with similar biomechanical gait patterns, constitutions, and load weights, which potentially mimic the possible effect of other habitual factors (like gait speed or ground reaction force) on gait. The second mechanism may be attributed to physiological, rather than biomechanical, responses to heavier loads, as confirmed and highlighted in other studies [24,25]. From a biomechanical perspective, carrying a heavy load near the center of gravity represents the most efficient method, as it minimizes energy consumption [26]. Physiologically, previous studies have shown that an increase in load of 15% may gradually increase both resting and active energy consumption by 5–6%, due to the trunk being positioned more forward compared to the no-load condition [25].

Despite the negative findings, this study is the first to investigate both spatial and temporal gait parameters in a representative sample of intervention police officers. Although the load increments did not affect the gait patterns, the findings of this study may have practical implications for examining the movement patterns of the arms, trunk, hips, and knees, providing more detailed information regarding the various angles and angular velocity properties of the joints. Additionally, the non-significant biomechanical discrepancies in spatial and temporal gait parameters should be interpreted through the lens of physiological mechanisms and 3-D kinematic and kinetic analyses, which would offer better insight into the factors contributing to these patterns.

This study has several limitations. Due to its cross-sectional design, we cannot determine causal relationships regarding the asymmetries, which limits the generalizability of the findings to police recruits, who have not yet gained sufficient experience with police tasks and equipment. Second, we only examined spatiotemporal gait parameters, while 3-D kinematic and electromyography systems would have provided additional insights into the increased gait asymmetries following the application of “a 3.5 kg/7.7 lb load”. Third, we did not assess biological and physiological parameters, which may clarify the relationship between the dynamic foot parameters and load carriage. Additionally, we did not gather data regarding injury history or the methods of load carriage, which limits our ability to draw practical implications for repositioning load items and exploring the potential effects of load carriage on injury incidence. Fifth, previous studies have shown different systematic evaluations of the feature encoding techniques of sensory data [27], like codebook-based and deep learning-based approaches. However, the same study confirmed that handcrafted feature-based techniques achieved a high recognition rate of approximately 96.0% for the recognition results of different human movements, showing such approaches to be as relevant as other new and more sophisticated techniques in presenting the data [27]. Finally, the participants walked barefoot over the pressure platform, which may have affected their gait patterns. Therefore, future research that is aimed at examining gait asymmetries during load carriage should focus on longitudinal study designs and comprehensive physiological and biomechanical analyses, as well as load- and injury-related characteristics. These factors may be crucial in limiting the negative effects of load carriage on the gait.

## 5. Practical Implications

Insignificant differences between the asymmetry of the spatial and temporal parameters of the gait using heavier equipment indicate how the members of the intervention police, as subjects of this research, achieved an established biomechanical pattern of movement in dynamic conditions. Namely, it is to be expected that asymmetries between the right and left side of the body will gradually increase, which was not the case in this study. From a practical point of view, it was determined that the different levels of equipment did not significantly affect asymmetry, which can be explained by learned motor control and ways of carrying the load itself during training or special tasks. However, there is still an unknown regarding the biomechanical differences between the right and left side of the body during long-term walking or running, which we could not confirm in this research. According to Knapik et al. [2] and Boffey et al. [5], the physiological component of carrying an external load can be more influenced than the biomechanical one, especially in populations that are subjected to the same or similar loads on a daily basis. Namely, it has been shown that the consumption of energy and oxygen increases physiologically during a heavy load, and the state of fatigue increases significantly [2,5]. On the other hand, fatigue could also be measured by the time spent walking across the platform with different loads, but due to the nature of the data collection and the daily activities of the emergency police personnel, this was not possible. Nevertheless, walking at a normal and habitual pace with heavier equipment did not affect the biomechanics of the lower extremities, but other components, mentioned in the Discussion Section, need to be further explored.

## 6. Conclusions

In summary, this study demonstrates that a heavier load carriage did not progressively increase spatial and temporal gait asymmetries in professional intervention police officers. Health and law enforcement personnel can benefit from these findings, as carrying heavy loads does not adversely affect body equilibrium or the disproportion of the gait between the left and the right sides of the body. However, other bodily functions, such as physiological changes during walking with heavier loads, should be examined and integrated into the system to identify the most significant factors influencing gait asymmetry.

## Figures and Tables

**Table 1:** Changes in the spatial gait parameters under the different loading conditions.

Study Variables	Left Foot	Right Foot	Mean Difference	Symmetry Index	*p*	η2
Spatial Gait Parameters	Mean (SD)	Mean (SD)				
Foot rotation (°) *						
No load	8.3 (4.9–11.4)	10.1 (7.4–14.7)	1.8	0.22		
Load 1	7.8 (4.9–11.4)	9.9 (6.1–14.4)	2.1	0.27		
Load 2	8.6 (5.2–11.6)	10.3 (7.7–14.0)	1.7	0.20		
Load 3	8.1 (5.3–10.7)	9.9 (6.7–13.9)	1.8	0.22	0.908	0.002
Step length (cm)						
No load	68.5 (5.6)	67.6 (5.9)	0.9	−0.02		
Load 1	68.7 (6.3)	68.7 (5.8)	0.0	0.00		
Load 2	68.5 (6.3)	68.5 (6.0)	0.0	0.00		
Load 3	68.9 (6.4)	69.0 (6.2)	0.1	0.00	0.424	0.009
Step width (cm)						
No load	15.3 (2.9)		/	/		
Load 1	15.4 (2.7)		/	/		
Load 2	15.6 (2.8)		/	/		
Load 3	15.7 (3.0)		/	/	0.759	0.003
Length of gait line (mm)						
No load	239.1 (26.3)	242.4 (18.2)	3.3	0.01		
Load 1	242.4 (22.1)	239.5 (23.6)	2.9	−0.01		
Load 2	245.1 (17.9)	240.9 (24.9)	4.2	−0.02		
Load 3	242.7 (22.9)	243.3 (19.4)	0.6	0.01	0.160	0.014
Single limb support line (mm)						
No load	121.6 (21.3)	125.7 (13.0)	4.1	0.03		
Load 1	127.1 (20.4)	122.0 (15.4)	5.1	−0.04		
Load 2	124.8 (13.9)	120.6 (17.2)	4.2	−0.03		
Load 3	123.5 (13.8)	120.7 (14.6)	2.8	−0.02	0.090	0.020

* denotes using median and interquartile range (25th–75th percentile); *p* < 0.05.

**Table 2:** Changes in the temporal gait parameters under the different loading conditions.

Study Variables	Left Foot	Right Foot	Mean Difference	Symmetry Index	*p*	η2
Temporal Gait Parameters	Mean (SD)	Mean (SD)				
Step time (s)						
No load	0.55 (0.04)	0.55 (0.04)	0.00	0.00		
Load 1	0.54 (0.04)	0.55 (0.06)	−0.01	0.02		
Load 2	0.54 (0.04)	0.55 (0.05)	−0.01	0.02		
Load 3	0.53 (0.04)	0.54 (0.04)	−0.01	0.02	0.576	0.006
Stance phase (%)						
No load	62.1 (2.1)	62.3 (1.7)	0.02	0.00		
Load 1	62.3 (1.9)	61.6 (3.1)	−0.7	−0.01		
Load 2	62.7 (1.8)	62.5 (1.9)	−0.2	0.00		
Load 3	62.8 (1.9)	62.5 (1.9)	−0.3	−0.01	0.140	0.017
Load response (%)						
No load	12.3 (1.5)	12.0 (1.9)	−0.3	−0.02		
Load 1	11.8 (1.6)	12.1 (1.5)	0.3	0.03		
Load 2	12.4 (1.9)	12.7 (2.0)	0.3	0.02		
Load 3	12.6 (1.5)	12.7 (2.2)	0.1	0.01	0.135	0.017
Mid-stance (%)						
No load	37.8 (1.7)	37.5 (3.9)	−0.3	−0.01		
Load 1	38.4 (3.0)	37.5 (2.0)	−0.9	−0.02		
Load 2	37.7 (2.3)	37.2 (2.0)	−0.5	−0.01		
Load 3	37.5 (2.1)	37.2 (2.0)	−0.3	−0.01	0.874	0.002
Pre-swing (%)						
No load	12.1 (1.9)	12.3 (1.5)	0.2	0.02		
Load 1	12.3 (1.6)	12.2 (2.0)	−0.1	−0.01		
Load 2	12.5 (2.0)	12.9 (1.8)	0.4	0.03		
Load 3	12.7 (2.1)	12.7 (1.4)	0.0	0.00	0.318	0.011
Swing phase (%)						
No load	37.9 (2.1)	37.7 (1.7)	−0.2	−0.01		
Load 1	37.6 (1.5)	38.3 (2.9)	0.7	0.02		
Load 2	37.3 (1.8)	37.5 (1.9)	0.2	0.01		
Load 3	37.2 (1.9)	37.5 (1.9)	0.2	0.01	0.161	0.016
Gait speed (km/h)						
No load	4.4 (0.5)		/	/		
Load 1	4.6 (0.5)		/	/		
Load 2	4.6 (0.6)		/	/		
Load 3	4.7 (0.6)		/	/	0.064	0.022

*p* < 0.05.

**Table 3:** Changes in the ground reaction force asymmetries under the different loading conditions.

Study Variables	Left Foot	Right Foot	Mean Difference	Symmetry Index	*p*	η2
Temporal Gait Parameters	Mean (SD)	Mean (SD)				
Forefoot (N)						
No load	852.3 (109.9)	865.6 (113.8)	13.3	0.01		
Load 1	873.0 (166.0)	893.0 (126.7)	20.0	1.79		
Load 2	960.6 (115.1)	967.6 (115.2)	7.0	0.01		
Load 3	978.4 (108.9)	984.4 (114.2)	6.0	0.01	0.151	0.016
Midfoot (N)						
No load	170.6 (70.3)	173.9 (68.7)	3.3	0.08		
Load 1	170.0 (74.2)	178.1 (75.4)	8.1	0.27		
Load 2	187.8 (75.4)	202.1 (81.4)	14.3	0.13		
Load 3	191.9 (82.6)	206.6 (82.0)	14.7	0.17	0.251	0.013
Hindfoot (N)						
No load	588.6 (89.9)	568.1 (84.5)	−20.5	−0.04		
Load 1	609.5 (82.0)	580.5 (94.0)	−29.0	−0.06		
Load 2	651.3 (86.6)	617.4 (87.3)	33.9	−0.06		
Load 3	662.2 (90.4)	636.6 (95.1)	−25.6	−0.03	0.323	0.011

## Data Availability

The raw data supporting the conclusions of this article will be made available by the authors on request.

## References

[1] Knapik J., Harman E., Reynolds K. (1996). Load carriage using packs: A review of physiological, biomechanical and medical aspects. Appl. Ergon..

[2] Knapik J.J., Reynolds K.L., Harman E. (2004). Soldier load carriage: Historical, physiological, biomechanical, and medical aspects. Mil. Med..

[3] Salvendy G. (2012). Handbook of Human Factors and Ergonomics.

[4] Larsen L.B., Tranberg R., Ramstrand N. (2016). Effects of thigh holster use on kinematics and kinetics of active duty police officers. Clin. Biomech..

[5] Boffey D., Harat I., Gepner Y., Frosti C.L., Funk S., Hoffman J.R. (2019). The Physiology and Biomechanics of Load Carriage Performance. Mil. Med..

[6] Faghy M.A., Shei R.J., Armstrong N.C.D., White M., Lomax M. (2022). Physiological impact of load carriage exercise: Current understanding and future research directions. Physiol. Rep..

[7] Walsh G.S., Low D.C. (2021). Military load carriage effects on the gait of military personnel: A systematic review. Appl. Ergon..

[8] Seeley M.K., Umberger B.R., Shapiro R. (2008). A test of the functional asymmetry hypothesis in walking. Gait Posture.

[9] Lanshammar K., Ribom E.L. (2011). Differences in muscle strength in dominant and non-dominant leg in females aged 20–39 years--a population-based study. Phys. Ther. Sport.

[10] Shi P., Fang Y., Guo M., Yu H. (2015). The effects of asymmetrical loading on gait characteristics. Biomed. Tech..

[11] Zhang X.A., Ye M., Wang C.T. (2010). Effect of unilateral load carriage on postures and gait symmetry in ground reaction force during walking. Comput. Methods Biomech. Biomed. Engin.

[12] DeVita P., Hong D., Hamill J. (1991). Effects of asymmetric load carrying on the biomechanics of walking. J. Biomech..

[13] Park K., Sy J.F., Horn G.P., Kesler R.M., Petrucci M.N., Rosengren K.S., Hsiao-Wecksler E.T. (2018). Assessing gait changes in firefighters after firefighting activities and while carrying asymmetric loads. Appl. Ergon..

[14] Majumdar D., Pal M.S., Pramanik A., Majumdar D. (2013). Kinetic changes in gait during low magnitude military load carriage. Ergonomics.

[15] Ozgül B., Akalan N.E., Kuchimov S., Uygur F., Temelli Y., Polat M.G. (2012). Effects of unilateral backpack carriage on biomechanics of gait in adolescents: A kinematic analysis. Acta Orthop. Traumatol. Turc..

[16] Štefan A., Kasović M., Štefan L. (2024). Does a Standardized Load Carriage Increase Spatiotemporal Gait Asymmetries in Police Recruits? A Population-based Study. Mil. Med..

[17] Kasović M., Štefan L., Borovec K., Zvonař M., Cacek J. (2020). Effects of Carrying Police Equipment on Spatiotemporal and Kinetic Gait Parameters in First Year Police Officers. Int. J. Environ. Res. Public Health.

[18] Teyhen D.S., Shaffer S.W., Goffar S.L., Kiesel K., Butler R.J., Rhon D.I., Plisky P.J. (2020). Identification of Risk Factors Prospectively Associated with Musculoskeletal Injury in a Warrior Athlete Population. Sports Health.

[19] Yavnai N., Bar-Sela S., Pantanowitz M., Funk S., Waddington G., Simchas L., Svorai-Litvak S., Steinberg N. (2021). Incidence of injuries and factors related to injuries in combat soldiers. BMJ Mil. Health.

[20] Sharma J., Weston M., Batterham A.M., Spears I.R. (2014). Gait retraining and incidence of medial tibial stress syndrome in army recruits. Med. Sci. Sports Exerc..

[21] Kasović M., Rožac D., Štefan A., Štefan L., Milković S. (2024). Effects of Different Load Carriage on Spatiotemporal Gait Parameters in Elite Intervention Police Officers. Appl. Sci..

[22] Robinson R.O., Herzog W., Nigg B.M. (1987). Use of force platform variables to quantify the effects of chiropractic manipulation on gait symmetry. J. Manip. Physiol. Ther..

[23] Maines J.M., Reiser R.F. (2006). Ground reaction force bilateral asymmetries during submaximal sagittal plane lifting from the floor. Int. J. Industr Ergon..

[24] Stuempfle K.J., Drury D.G., Wilson A.L. (2004). Effect of load position on physiological and perceptual responses during load carriage with an internal frame backpack. Ergonomics.

[25] Quesada P.M., Mengelkoch L.J., Hale R.C., Simon S.R. (2000). Biomechanical and metabolic effects of varying backpack loading on simulated marching. Ergonomics.

[26] Heglund N.C., Willems P.A., Penta M., Cavagna G.A. (1995). Energy-saving gait mechanics with head-supported loads. Nature.

[27] Fatima R., Khan M.H., Nisar M.A., Doniec R., Farid M.S., Grzegorzek M. (2024). A Systematic Evaluation of Feature Encoding Techniques for Gait Analysis Using Multimodal Sensory Data. Sensors.

